# Dual Action of Mexiletine and Its Pyrroline Derivatives as Skeletal Muscle Sodium Channel Blockers and Anti-oxidant Compounds: Toward Novel Therapeutic Potential

**DOI:** 10.3389/fphar.2017.00907

**Published:** 2018-01-12

**Authors:** Michela De Bellis, Francesca Sanarica, Alessia Carocci, Giovanni Lentini, Sabata Pierno, Jean-François Rolland, Diana Conte Camerino, Annamaria De Luca

**Affiliations:** ^1^Unit of Pharmacology, Department of Pharmacy-Drug Science, University of Bari Aldo Moro, Bari, Italy; ^2^Unit of Medicinal Chemistry, Department of Pharmacy-Drug Science, University of Bari Aldo Moro, Bari, Italy; ^3^AXXAM S.p.A., Milan, Italy

**Keywords:** voltage-clamp recordings, sodium channel, skeletal muscle, anti-myotonic agent, muscular dystrophy, mexiletine, C_2_C_12_ cells, antioxidant action

## Abstract

Mexiletine (Mex) has been recently appointed as an orphan-drug in myotonic-syndromes, being a potent use-dependent blocker of skeletal-muscle sodium channels (Na_V_1.4). Available evidences about a potential anti-oxidant effect of Mex and its tetramethyl-pyrroline-derivatives *in vivo*, suggest the possibility to further enlarge the therapeutic potential of Mex-like compounds in myopathies in which alteration of excitation-contraction coupling is paralleled by oxidative stress. In line with this and based on our previous structure-activity-relationship studies, we synthesized new compounds with a tetramethyl-pyrroline-ring on the amino-group of both Mex (VM11) and of its potent use-dependent isopropyl-derivative (CI16). The compounds were tested for their ability to block native Na_V_1.4 and to exert cyto-protective effects against oxidative-stress injury in myoblasts. Voltage-clamp-recordings on adult myofibers were performed to assess the tonic and use-dependent block of peak sodium-currents (I_Na_) by VM11 and CI16, as well as Mex, VM11 and CI16 were 3 and 6-fold more potent than Mex in producing a tonic-block of peak sodium-currents (I_Na_), respectively. Interestingly, CI16 showed a 40-fold increase of potency with respect to Mex during high-frequency stimulation (10-Hz), resulting the strongest use-dependent Mex-like compound so far. The derivatives also behaved as inactivated channel blockers, however the voltage dependent block was modest. The experimental data fitted with the molecular-modeling simulation based on previously proposed interaction of main pharmacophores with Na_V_1.4 binding-site. CI16 and VM11 were then compared to Mex and its isopropyl derivative (Me5) for the ability to protect C_2_C_12_-cells from H_2_O_2_-cytotoxicity in the concentration range effective on Na_v_1.4. Mex and Me5 showed a moderate cyto-protective effect in the presence of H_2_O_2_, Importantly, CI16 and VM11 showed a remarkable cyto-protection at concentrations effective for use-dependent block of Na_V_1.4. This effect was comparable to that of selected anti-oxidant drugs proved to exert protective effect in preclinical models of progressive myopathies such as muscular dystrophies. Then, the tetramethyl-pyrroline compounds have increased therapeutic profile as sodium channel blockers and an interesting cyto-protective activity. The overall profile enlarges therapeutic potential from channelopathies to myopathies in which alteration of excitation-contraction coupling is paralleled by oxidative-stress, i.e., muscular dystrophies.

## Introduction

Mexiletine (Mex) is an antiarrhythmic drug belonging to class IB used in the treatment of arrhythmias consequent to myocardial ischemia, its activity being related to the use-dependent inhibition of the rapid inward sodium currents. However, clinical trials have confirmed the usefulness of this drug in many pathological conditions. In particular, mexiletine controls hyperexcitability in both dystrophic and non-dystrophic myotonias (Logigian et al., 2010; Hoffman and Kaminski, 2012) and relieves neuropathic pain (Tremont-Lukats et al., 2005). Mex has also been proposed as a novel therapeutic approach in Timothy syndrome (Gao et al., 2013) and to successfully treat lidocaine-responsive neonatal epilepsy (Nakazawa et al., 2013). In addition, a very recent study has reported, for the first time, that Mex is able to reduce the occurrence of arrhythmic events in LQT3 patients, including infants (Mazzanti et al., 2016). Importantly, mexiletine, and some related compounds have been described to be protective against brain anoxic injury and against the ischemia reperfusion-induced oxidative myocardial damage (Li et al., 2000; Hewitt et al., 2001; Chang et al., 2002; Halmosi et al., 2002). This protection can be related to the blockage of sodium channels resulting in turn in the inhibition of Na^+^-Ca^2+^ exchanger-dependent Ca^2+^ overload. However, a favorable antioxidant property can also play a role (Shankar et al., 2000; Wagner et al., 2011). In particular, Demirpence et al. showed that mexiletine inhibits free-radical-induced lipid peroxidation in brain membranes, as well as in the liver microsomes, probably as a result of a direct interaction of the drug with the biological membranes (Demirpençe et al., 1999). However, the available evidences suggest that the increase in the cardioprotective activity is obtained with the introduction of a 2,2,5,5,-tetramethyl-pyrroline moiety on the amino terminal group of mexiletine, likely in relation to an anti-oxidant effect of this moiety (Li et al., 2000; Shankar et al., 2000; Halmosi et al., 2002). Compounds with a dual action as use-dependent blockers and anti-oxidants may have an interesting therapeutic action in degenerating myopathies in which the alteration of excitation-contraction coupling is accompanied by chronic inflammatory state and unbalanced oxidative stress, such as Duchenne Muscular Dystrophy (DMD) (De Luca et al., 2008; Burdi et al., 2009), caused by defects in the subsarcolemmal protein dystrophin, a component of the dystrophin-glycoprotein complex (DGC) (De Luca et al., 2012). In particular, voltage-gated skeletal muscle sodium channels (Na_V_1.4) are physically linked to the DGC via α-syntrophin (Koenig et al., 2011). The absence of dystrophin and the disorganization of the complex leads to controversial alterations in Na_V_1.4 channel biophysics and expression, which can be also worsened, either directly or indirectly, by oxidative stress (Gavillet et al., 2006; Hirn et al., 2008; Pal et al., 2014; Choi et al., 2016). This latter can also lead to inefficient myogenic program and repair after injury, due to a direct damage of myogenic precursors (Kozakowska et al., 2015). In addition, our previous electrophysiological experiments showed an increased excitability in acutely degenerating muscle of dystrophic mdx mouse model of DMD, due to impaired chloride channel conductance (De Luca et al., 1997a). This may increase the mechanical stress on weak dystrophin-deficient myofibers, and reinforces the interest of sodium channel blockers as potential therapeutics.

Our previous structure-activity relationship studies have provided key information about structural requirements of mexiletine-like compounds for enhancing the use-dependent block of Na_V_1.4 channels. In particular, we disclosed a key role of substituents on the chiral center nearby the terminal amino group, such as in the potent Me5 bearing an isopropyl group on the chiral center of Mex backbone (De Bellis et al., 2006, 2013). In this frame, the present study combined voltage-clamp recordings in native striated myofibers and assessment of C_2_C_12_ myoblasts viability in order to evaluate (a) if mexiletine and its potent derivative Me5 exert a cyto-protective action against the oxidative stress-induced damage of myogenic progenitors in the same concentration range they block Na_V_1.4 channels; (b) the effect of the introduction of a tetramethyl-pyrroline moiety on the amino-group of Mex (as in VM11) and of Me5 (as in CI16), on both the use-dependent block of Na_V_1.4 channels and cyto-protective profile. This latter test was performed in parallel with selected compounds with anti-oxidant properties of interest for muscular dystrophy, as *N*-acetylcisteine (NAC) and PP2.

The results showed that the introduced structural modification greatly enhanced therapeutically relevant use-dependent and cyto-protective actions. This paves the way to more addressed *in vivo* studies in degenerating myopathies as well as to a rational design of novel class of dually acting compounds with wider therapeutic indications.

## Materials and methods

The screening of compounds on I_Na_ of single muscle fibers was carried out by means of voltage-clamp recordings based on methods described by Hille and Campbell (1976).

### Voltage-clamp recordings

Segments of undamaged single muscle fibers (about 1 cm in length and 80 μm in diameter) were obtained by microsurgery (plucking procedure) from the ventral branch of the semitendinosus muscle of Rana Esculenta and bathed in normal physiological solution at room temperature. The cut-end fiber was then bathed in an internal solution and mounted across three chamber partitions, which delineated four pools. Three strips of vaseline were applied over the fiber and carefully sealed to the fiber to reduce leakage. The width of the gaps of the central pools (A and B) had been previously set to 70–100 μm and 200 μm, respectively. Four KCl/agar bridges electrodes connected the recording chamber to the voltage-clamp amplifier based on methods described by Hille and Campbell (1976). For recording sodium currents (I_Na_), the solution in the pool A was replaced with the external solution and after about 10 min of equilibration the recordings were performed at 10°C. The usual holding potential (H.P.) was−100 mV, unless otherwise specified (De Luca et al., 2012).

The mean value of membrane area in this pool, from which sodium currents were recorded, was 3.4 ± 0.8 × 10^−4^ cm^2^ (*n* = 40). Particular care was taken to verify that no change occurred in the membrane area in pool A throughout the experiments. Sodium currents were recorded using an amplifier connected via a A/D and D/A Digidata 1,200 Interface (Axon) to a 486 DX2/66 personal computer and stored on the hard disk. The stimulation protocols and data acquisition were controlled by Clampex software (pClamp6; Axon Instruments, Foster City, CA, USA) (Mele et al., 2014). The currents flowing in response to depolarizing command voltages were low pass filtered at 10 kHz (Frequency Devices, Haverhill, MA, USA), visualized on an oscilloscope and sampled at 20 kHz. When necessary, leak and capacities currents were subtracted by P/4 method. The acquired traces were analyzed offline using Clampfit software (pClamp6; Axon Instruments). This method was shown to be predictable of drug efficacy on mammalian sodium channel and myotonic conditions (De Luca et al., 2004; De Bellis et al., 2017a).

### Solutions and drugs

The following solutions were used: normal physiological solution, 115 mM NaCl, 2.5 mM KCl, 1.8 mM CaCl_2_, 2.15 mM Na_2_HPO_4_×12H_2_O, 0.85 mM NaH_2_PO_4_×H_2_O (pH = 7.3); external solution, 77 mM NaCl, 2.5 mM KCl, 38 mM Choline-Cl, 1.8 mM CaCl_2_, 2.15 mM Na_2_HPO_4_×12H_2_O, 0.85 mM NaH_2_PO_4_×H_2_O (pH = 7.3); internal solution, CsF 105 mM, MOPS sodium salt 5 mM, MgSO_4_ 2 mM, EGTA 5 mM, Na_2_ATP 0.55 mM (the pH was set to 7.2 with NaOH).

The compounds tested and shown in Table 1 were 2-(2,6-dimethylphenoxy)-1-amino-propanemethylethylamine (Mex); 1-(2,6-dimethylphenoxy)-3-methyl-2-butanamine (Me5); the Mex-derivative 2,2,5,5-tetramethyl-N-[1-methyl-2-(2,6-dimethylphenoxy)ethyl]-2,5-dihydro-1H (VM11); the Me5-derivative 2,2,5,5-tertramethyl-N-[1-(2,6-dimethylphenoxy)-3-methylbutan-2-ethyl]-2,5-dihydro-1H-pyrrole-3 carboxamide (CI16). The abbreviated nomenclature used throughout the text was assigned at the time the compounds were synthesized and is, therefore, arbitrary. Mexiletine was purchased from Sigma-Aldrich (Milan, Italy).

**Table 1:** Chemical structures and physicochemical parameters of Mex and its tetramethyl-pyrroline derivatives.

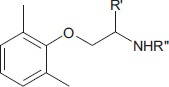					
**Compound**	**R′**	**R″**	**Log** ***P***	**p*K_a_***	**Log *D*_7.4_**
Mex	CH_3_	H	2.2 ± 0.01	9.28 ± 0.01	0.3 ± 0.1
Me5	CH_3_-CH-CH_3_	H	2.72 ± 0.02	8.97 ± 0.03	1.1 ± 0.1
VM11	CH_3_	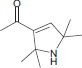	2.8 ± 0.1	8.5 ± 0.1	1.7 ± 0.1
CI16	CH_3_-CH-CH_3_	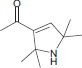	3.2 ± 0.3	8.53 ± 0.01	2.1 ± 0.1

*Mex; 1-(2,6-dimethylphenoxy)-3-methyl-2-butanamine (Me5); N-[2-(2,6-dimethylphenoxy)-1-methylethyl]-2,2,5,5-tetramethyl-2,5-dihydro-1H-pyrrole-3-carboxamide (VM11); N-{1-[(2,6-dimethylphenoxy)methyl]-2-methylpropyl}-2,2,5,5-tetramethyl-2,5-dihydro-1H-pyrrole-3-carboxamide (CI16); pKa ionization costants. The pKa values refer to the amine group of the drugs. LogP and pKa values were obtained as described in Materials and Methods; LogD_7.4_ values were calculated from LogP and pKa values*.

The 2,2,5,5-tetramethyl-pyrrolidine-3-pyrroline-3-carboxamides *R*-VM11, *S*-VM11, and *RS*-C116 were prepared from the corresponding amines *R*-Mex, *S*-Mex, and *RS*-Me5, respectively, following a previously reported procedure (Hankovszky et al., 1986). In turn, Mex enantiomers were obtained as previously reported (Carocci et al., 2000; Catalano et al., 2004), while *RS*-Me5 was obtained adapting a recently developed procedure (Carocci et al., 2000). Physicochemical features and synthetic details will be reported elsewhere (Gualdani et al., 2015).

Stock solution in external solution containing dimethyl sulfoxide (DMSO, <1%) were used for the VM11 and compound CI16. DMSO at the highest concentration used for dilution (0.2%) was without effect on the parameters recorded. Drugs were readily soluble in the physiological solution; fresh stock solution was daily made and diluted as required. The pH of the drug-containing solutions was carefully monitored to be within the physiological range of 7.2–7.4; however, no pH correction was necessary.

### Pulse protocols analysis

The tonic block (TB) exerted by each compound was calculated as the percent reduction of the maximal peak sodium transient (I_Na, max_) elicited by infrequent depolarizing steps to −20 mV from the holding potential (HP) of −100 mV, close to the physiological potential, at a frequency of 0.3-Hz. The use-dependent block (UDB) exerted by each drug was evaluated using trains of 10 ms test pulses from the HP to −20 mV, applied at stimulation frequencies from 0.3 up to 10-Hz for 30 s. The use-dependent behavior of test compounds was estimated by the further reduction of I_Na_ that progressively increased above the tonic block in a frequency-dependent manner until a new steady-state amplitude was reached. The degree of use-dependent block was computed as the value of the steady-state I_Na, max_ at this point normalized to the current at the same point in the absence of drug. This parameter reflects the potency of the drug for blocking the channels under conditions of high-frequency stimulation (i.e., as occurs during myotonic action potential firing) (De Luca et al., 2003a,b). The voltage-dependent block was obtained using a pulse protocol of infrequent depolarizing stimulation (0.3-Hz) to −20 mV, for 10 ms from two different HP values: −140 mV (very negative to have all channels in the resting state), and −70 mV more depolarized to resemble a pathological condition, in which a proportion of sodium channels are inactivated (De Bellis et al., 2006). The concentration-dependence of the drug effect was calculated as the half-maximal blocking concentration (IC_50_) at −140 mV, which allowed calculation of the affinity constant for the resting state (K*r*). The IC_50_ value calculated from a HP of −70 mV is influenced both by the higher proportion of channels entering a closed-state inactivation at this potential and by the possible ability of the drug, if acting as an inactivated channel blocker, to modify this distribution in favor of more inactivated channels. The affinity constant of the drug for the inactivated channel (K_i_) was the computed as described below.

Steady-state inactivation (h∞) curves were determined by a cyclic protocol of pulse sequences. Each sequence consisted of a conditioning pulse to −140 mV, a prepulse of 1000 ms duration, and a test pulse to −20 mV for 10 ms; after a pause of 3 s, the sequence was repeated 18–20 times with the prepulse potential value increased each time in 5 mV steps. The estimate of the affinity constant for the inactivated state (K_i_) was obtained from the voltage dependent distribution of the channels in the resting (h) and inactivated state (1-h) obtained from the h∞ curve, using the equation 1/K_−70_ = h/K_r_ (1-h)/K_i_, where K_−70_ and K_r_ are the IC_50_ values obtained from dose-response curves at −140 and −70 mV, and h and (1-h) represent the fraction of channels in the resting and inactivated state at −70 mV, respectively (De Luca et al., 2012; Muraglia et al., 2014).

Recovery from inactivation was measured as residual current at the end of trains of pulses at decreasing frequency from 10- to 0.25-Hz, in the absence and presence of drug. The recovery occurred as a mono-exponential process for the compounds; each point has been obtained by normalizing the residual current at the end of each train (equilibrium) to the I_Na, max_ obtained at the longest interpulse used (0.25-Hz). The values have been plotted against the duration of the interval between pulse and fitted to a single exponential. Time constant values have been obtained from the fit described above and have been plotted against the concentrations of each compound (De Bellis et al., 2006).

### Cell culture cytotoxicity assay

Murine C_2_C_12_ skeletal muscle cells were cultured in DMEM supplemented with 10% fetal bovine serum, 1% penicillin, 1% streptomycin and 1% glutamine and were maintained at 37°C in 5% CO_2_/95% air. Cells obtained by sub-confluent cultures (about 70%) were seeded in 96 multiwell plate at a density of approximately 4.5 × 10^3^ cells per well. Eighteen hours after seeding, either the cytotoxic effect of H_2_O_2_, mexiletine, Me5, their tetramethyl-pyrroline derivatives, and PP2 or the potential protective effect of all above mentioned compounds and N-acetyl cysteine (NAC) against the H_2_O_2_-induced oxidative stress has been investigated. The cytotoxic effect of increasing concentration of H_2_O_2_ (Sigma Aldrich), Mex and its analogs, and PP2 was tested on C_2_C_12_ cells after 8 h of incubation. To evaluate the cyto-protective effect of the drugs, C_2_C_12_ cells were incubated with the test compounds 2 h before the application of H_2_O_2_ at 300 μM and 1 mM, and then maintained for additional 6h. Two hours before absorbance lecture, 10 μl of Cell Counting Kit-8 pure solution (CCK-8 Alexis Biochemicals) was added into each well. The CCK-8 solution contains the monosodium salt of [2-(2-methoxy-4-nitrophenyl)-3-(4-nitrophenyl)-5-(2,4-disulfophenyl)-2H-tetrazolium (WST-8), which is reduced by cellular dehydrogenases to give a yellow colored product (formazan) absorbing at 450 nm. The quantity of formazan dye is directly proportional to the number of viable cells. Absorbance was measured at 450 nm with a microplate spectrophotometer (Victor V31420-40, PerkinElmer). This assay was successfully used to investigate on the mechanism of ion channel-regulated cell proliferation in cell lines (Tricarico et al., 2013).

Fresh stock solutions of the test compounds were made daily and diluted in DMEM as required. Only for the tetramethyl-pyrroline derivatives and PP2, stock solutions in DMSO were prepared and then diluted in DMEM. The maximal concentration of DMSO in the diluted solutions was <0.2%. As expected, 0.2% DMSO had no effect on cell survival (data not shown). Accordingly, cell viability in the presence of drugs was expressed as percent change with respect to the cell treated with vehicle alone, after proper subtraction of blank absorbance. The drug-free wells had comparable values of absorbance between the plates (1.325 ± 0.021; *n* = 31).

### Data analysis and statistics

All data were expressed as mean ± S.E.M. The IC_50_ values, in the various experimental conditions, were determined by using a non-linear least squares fit of the concentration-response curves to the following logistic equation: Effect = −100/{1+(K/[drug])n}, where Effect is the percentage change of I_Na_; −100 is the maximal percentage block of I_Na_; K is IC_50_; n is the logistic slope factor, and [drug] is the molar concentration of the compound (Talon et al., 2001). A similar fitting was used to assess the IC_50_ for cytotoxic action on C_2_C_12_ cells, expressing the percent of viable cell with respect to the drug concentration tested. The h∞ curves have been fitted with a single Boltzmann, and the potential at which 50% of the sodium channels were inactivated (Vh_1/2_) was calculated at the inflection point of the curves. Statistical significance of differences between pairs of mean values has been estimated by unpaired Student's *t*-test and considered significant for *p* < 0.05. Two-way ANOVA and Bonferroni post-hoc corrections have been used for multiple comparison of experimental data means. The statistical significance between IC_50_ values obtained from the fit was evaluated using a number of degrees of freedom equal to the total number of preparations determining each point of the curve minus the number of means determining the curve minus two for the free parameters. The measurements of ionization constants and lipophilicity were performed on a Sirius GLpKa analyzer for pH-metric p*K*a and Log*P* (Sirius Analytical Instruments Ltd-Forest Row-UK). Correlation analysis was evaluated by fitting the experimental data point to linear regression analysis. Nonlinear equation fitting and processing for data graphics were done by FigP Software (Biosoft, Cambridge, UK).

### Quantum mechanical calculations

A previously reported procedure was adopted (De Bellis et al., 2013; Gualdani et al., 2015). The models of mexiletine (VM11) and its tetramethyl-pyrrolyl-carbonyl derivative (CI16) were generated from atomic fragments incorporated into Spartan'14 (Wavefunction Inc., Irvine, CA) inner fragment library and assuming the suggested default starting geometries. Protonated species were considered. The generated geometries were optimized by the molecular mechanics MMFF routine offered by the software (Halgren, 1996) and then submitted to a systematic conformational distribution analysis using the default step sizes. All conformers in a window of 10 Kcal/mol above the global minimum conformer were retained. When two conformers differed by dihedral values lower than 10°, the less stable conformer was left out. Conformers were then classified according to their ab initio gas phase energy content calculated at the RHF/3-21G^*^ level. All conformers falling within a window of 5 kcal/mol above global minimum were retained and submitted to RHF/3-21G^*^ geometry optimization. After removal of redundant conformers (i.e., each conformer differing from a more stable one by <5° in their corresponding dihedral values), the single point energy content for all the remaining conformers were calculated at the RHF/6-31G^**^ level. The optimized structures were confirmed as real minima by IR frequency calculation. The most stable conformer of each compound underwent geometry optimization by density-functional theory (DFT) implemented in Spartan'14 with B3LYP functional (Becke, 1988), and 6-31G^**^ basis set (Davidson and Feller, 1986) in the gas phase.

## Results pharmacology

Mexiletine and the analogs with structural modification at the level of the alkyl chain and of the chiral center and their physicochemical parameters are shown in Table 1.

### Block of sodium currents of the mexiletine and of tetramethyl-pyrroline derivatives

We first evaluated the effects of mexiletine on Na_V_1.4, in order to confirm its activity in the present experimental condition. In a manner consistent with previously published data (De Bellis et al., 2006), the use-dependent behavior was clearly detected for Mex, with a ratio (IC_50_ tonic block/IC_50_ 10-Hz use-dependent block) of 3.2 (Table 2). The profile of Mex was also accompanied by a certain degree of stereoselectivity, the *R*-enantiomer being 1.7 times more potent than the *S*-one in the tonic block (De Bellis et al., 2006). However, in line with our published data (Catalano et al., 2004; Carocci et al., 2010; De Bellis et al., 2013), when the stimulation frequency was increased, the stereoselectivity was attenuated, as expected by the long-time constant for recovery from inactivation of the blocked Na_V_1.4.

**Table 2:** Concentrations for half-maximal tonic and use-dependent block of I_Na_ by Mex, Me5, and their corresponding tetramethyl-pyrroline derivatives.

**Compound**	**Tonic block IC_50_ (μM)**	***R***	**Use-dependent block 10-Hz IC_50_ (μM)**	***R***	**BT/BUD**
**Mex**	75.3 ± 8	*1*	23.6 ± 2.8	*1*	3
*R*-Mex	74 ± 8^∞^		31 ± 8		2
*S*-Mex	127.0 ± 2.8		32 ± 7		4
*R*-Me5	25.2 ± 0.6^*^^∞^		5.7 ± 0.5^*^		4
*S*-Me5	46.1 ± 1.1		5.8 ± 0.6		8
**VM11**	23.4 ± 0.9^*^	*3.2*	2.0 ± 0.1^*^^#^	12	12
*R*-VM11	17.2 ± 2.4		1.1 ± 0.01		16
*S*-VM11	25 ± 4		2.2 ± 0.1		11
**CI16**	12.6 ± 0.2^*^^#^^◦^	*6*	0.6 ± 0.1^*^^#^^◦^	*39*	21

*Concentrations able to produce half-maximal response (IC_50_, μM) in producing a tonic block and a use-dependent block. The ratio between IC_50_ values during tonic and use-dependent block (IC_50_ TB/IC_50_ UDB 10-Hz) is shown in order to allow an easier comparison of the use-dependent behavior of each compound. The IC_50_ values have been obtained during non-linear least-square fit of the concentration-response data to the logistic equation described in Materials and Methods. The ratio (R) between IC_50_ values of Mex and IC_50_ values of each compound in racemic form, for the tonic and use-dependent block, is also shown to better evaluate the relative potency toward the parent compound. Each value is the mean ± S.E.M. from four to seven fibers*.

Statistical significance by Student's t-test (with p < 0.001 or less) is as follow:

* vs. Mex;

# vs. R-Me5;

◦ between the tetramethyl-pyrroline derivatives;

∞ *between the enantiomers of the same compound*.

In Table 2, the effects of Mex as both racemate and enantiomers, are compared to those of the Me5 derivative, in which the increased hindrance on the asymmetric carbon atom was obtained by replacing the methyl group of mexiletine with an isopropyl group. This structural change causes a marked increase in the potency for producing a tonic and a use-dependent block with respect to parent compound, likely in relation to a better interaction with the binding site in high affinity states (De Luca et al., 2000, 2012; Carocci et al., 2010).

**Figure 1:**
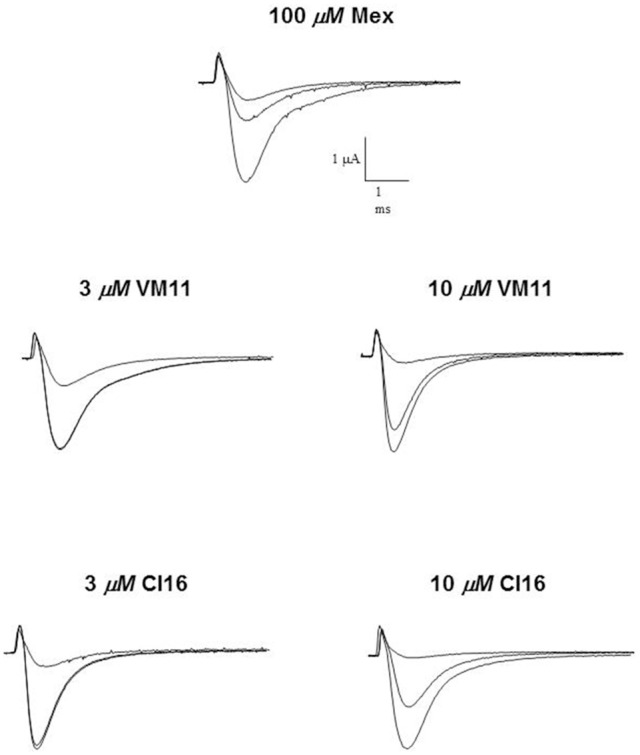
Na^+^ current transients recorded in the absence and in the presence of 100 μM Mex, 3–10 μM VM11 and 3–10 μM CI16. In each group of traces, the greatest one has been recorded in the absence of drug, with a depolarizing step from the HP of −100 to −20 mV for 10 ms. A similar depolarizing stimulus applied after the application of each compound allowed to estimate the tonic block exerted by the drug (middle traces). The smallest current traces correspond to the residue current at end of the 10-Hz stimulation protocol. In particular, at concentration 3 μM, for bothtetramethyl-pyrroline the trace of the tonic block is superimposed on that of the control.

**Figure 2:**
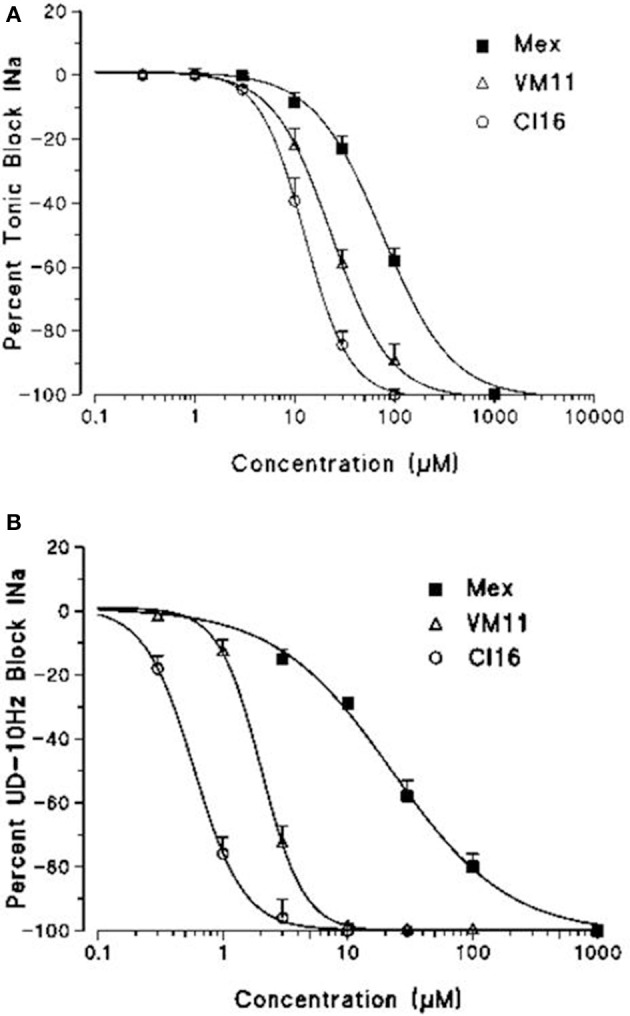
Concentration-response curves for tonic **(A)** and 10-Hz use-dependent block **(B)** of Na^+^ currents obtained with Mex and its tetramethyl-pyrroline derivatives VM11 and CI16. Each point shows the per cent block of I_Na_ observed in the presence of each concentration of drug vs. I_Na_ in the absence of the drug in the same fiber and is the mean ± S.E.M. from three to seven fibers the curves fitting the experimental points were obtained using the logistic function described in Materials and Methods.

We then assessed the activity of the new Mex and Me5 derivatives on Na_V_1.4 channel (De Bellis et al., 2017a). This was of importance considering the presence of the pyrroline moiety on the main pharmacophore group (amino-terminal group). Interestingly, the introduction of a tetramethyl-pyrroline moiety in Mex backbone, as in VM11, markedly enhanced the drug potency on the I_Na_. This can be appreciated in Figure 1 where the effects produced by both 3 μM and 10 μM VM11 are shown. In particular, VM11 was about 3-fold more potent than Mex in producing the tonic block (IC_50_ = 23.4 ± 0.9 μM) (Table 2). This increase in potency was not attributable to an increase in log*P* value (2.8 ± 0.1), the latter being similar to that of the parent compound (2.2 ± 0.01) (Table 1), thus suggesting the involvement of more specific chemical interactions of new analog at the binding site. VM11 behaved as a classical and potent use-dependent channel blocker (Table 2, Figure 2). The concentration-response curve constructed after a train of depolarizing pulses at the frequency of 10-Hz was markedly left-shifted compared to that of parent compound (Figure 2B). As can be seen in Table 2, the analog showed a markedly enhanced use-dependent behavior, with a ratio (IC_50_ TB/IC_50_ 10-Hz UDB) of 11.7. However, this trend is not correlated with an increased basicity since VM11 has a lower p*K*_a_ value (8.5 ± 0.1) than Mex (9.28 ± 0.01), and might be rather attributable to a more favorable logD value, in agreement with our published data (Table 2; De Bellis et al., 2013; Muraglia et al., 2014). Enantiomers of VM11 maintained the same behavior of the parent compounds, in term of attenuation of stereoselectivity during use-dependent blockade (Table 2).

**Figure 3:**
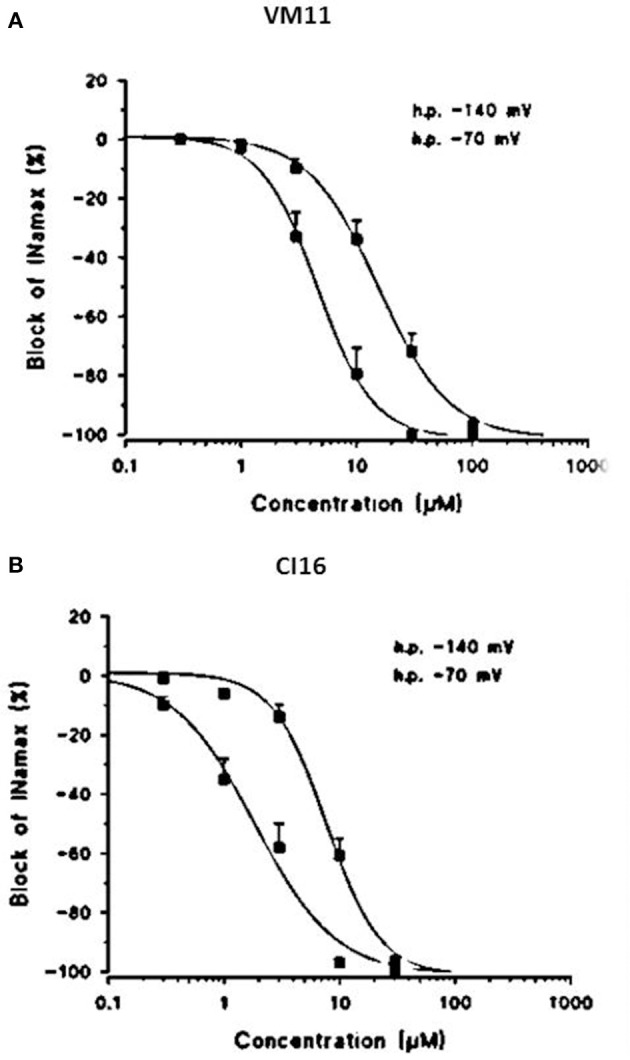
Concentration-response curves obtained with the tetramethyl-pyrroline derivatives VM11 **(A)** and CI16 **(B)**, constructed at the HPs values of −140 and at −70mV. The curves fitting the experimental points were obtained using the logistic function described in Materials and Methods and allowed the calculation of the IC_50_ values (in Table 3). Each value is the mean ± S.E.M. from four to seven fibers of the percentage block of I_Na_ in the absence of the drug in the same.

Subsequently, we assessed whether the introduction of a tetramethyl-pyrroline moiety on the amino group of the isopropyl derivative, as in CI16 (Table 1), may further increase the potency, with particular attention to the use-dependent activity. CI16, synthesized and available only as racemate, was about 6 and 40-fold more potent than mexiletine; and 2 and 9-fold more potent than Me5 (De Luca et al., 2000) in producing the tonic and the use-dependent block, respectively (Table 2). In agreement with the increased potency of this analog, the concentration-response curves were markedly left-shifted compared to those of Mex and VM11, for both tonic and 10-Hz use-dependent blocks, respectively (Figures 2A,B). Surprisingly, the new analog CI16 is the most use-dependent mexiletine-like sodium channel blocker described so far, with a ratio IC_50_ TB/IC_50_ 10-Hz UDB of 21 (Table 2). The use-dependent behavior is a complex dynamic process involving the kinetics of drug binding to and unbinding from the channel in relation to both state-dependent drug affinity and physicochemical properties (De Bellis et al., 2017a,b). The recovery from inactivation of drug-bound channels was determined. This process occurred with a mono-exponential time course for all the compounds tested and was clearly concentration dependent (De Bellis et al., 2006). CI16, that showed the highest use-dependent behavior, was also characterized by the slowest time constants. For instance, at both 3 and 10 μM, concentrations of both analogs exerting a use-dependent block of I_Na_ ranging between 60 and 70%, the time constants for recovery were significantly longer for CI16 with respect to that of VM11. As expected, the time constants of both compounds were significantly longer than those observed with Mex at the same concentrations (Figure S1). In view of the interesting use-dependent behavior of the two tetramethyl-pyrroline derivatives, we investigated their channel state-dependent affinity at two different HP, −140 and −70 mV, in conditions of low-frequency stimulation (0.3-Hz), which would be compared to the previously measured tonic block exerted at −100 mV. The scale of potency of the compounds for binding the resting channels (K_*r*_), evaluated at a HP of −140 mV was the same of that found for TB at −100 mV, being CI16 > VM11. However, due to the basic differences in the protocols used for assessing voltage dependent block and TB, and to the known influence of channel state in effect of sodium channel blockers, the apparent potency of VM11 and CI16 appeared to be slightly higher at −140 mV than at −100 mV. We considered this difference to be trivial. As expected from inactivated channel blockers (Talon et al., 2001), a great increase of potency was observed when the membrane potential was held at −70 mV; in fact, the concentration-response curves of the two tetramethyl-pyrroline derivatives were clearly shifted to the left with respect to those obtained at −140 mV (Figures 3A,B). CI16 was more potent than VM11 in blocking the channels in the inactivated state with a K_−70_ equal to 1.8 ± 0.4 μM (Table 3). However, the voltage-dependent block of both VM11 and CI16 was less important than that of Mex, suggesting that mechanisms other than inactivated channel block may account for the use-dependent behavior. We tested whether the two derivatives act as open channel blockers. In fact, no differences were observed in the drug-induced block calculated on the I_Na_ area with respect to that calculated at the I_Napeak_, nor a shift was found in the I/V curves for both compounds, allowing us to rule out an open channel blocking activity (data not shown).

**Table 3:** Voltage-dependent block of sodium currents by Mex and tetramethyl-pyrroline derivatives.

**Drug**	**Voltage-dependent block**		**Inactivated state block**
	**HP−140 mV IC_50_ (μM); K_r_**	**HP−70 mV IC_50_ (μM); K_−70_**	**Calculated K_i_ (μM)**
Mex	147.9 ± 20	1.9 ± 0.2	2
VM11	15.5 ± 0.6^*^#	4.5 ± 0.1^*^#	4.2
CI16	7.7 ± 0.5^*^s	1.8 ± 0.4^*^	1.8

*HP, holding potentials; IC_50_, half-maximal blocking concentration; K_i_, affinity constant for the inactivated state; Kr, affinity constant for the resting state. The columns from left to right are as follows: drug used; IC_50_ values at two different HP values: −140 and −70 mV; inactivated-state block refers to the affinity constants for inactivated sodium channels (K_i_) according to the equation in Methods. Each value is the mean ± S.E.M. from four to seven fibers*.

Statistical significance by Student's t-test (with p < 0.001 or less) is as follow:

* vs. Mex;

# *between the pyrroline derivatives*.

The two tetramethyl-pyrroline derivatives shifted the steady-state channel availability (h∞ curves) toward more negative potentials. In particular, between the two analogs, CI16, which was the most potent in blocking sodium channels, was also the most effective in shifting the h∞ curves toward more negative potentials. In fact, at concentration of 10 μM, this latter produced a shift in the h∞ curves (6.3 ± 1.1 mV, *n* = 5) almost twofold higher than that of VM11 (3.6 ± 1.1 mV, *n* = 4; *p* < 0.05) (Figures S2, S3). The results lead us to assume that the high use-dependent block is related to a high affinity binding to the channel in either resting and inactivated state, which lead to a slow unbinding kinetic, and finally to a progressive accumulation of blocked channel at high frequency stimulation.

### *Ab initio* molecular modeling of action of tetramethyl-pyrroline derivatives on Na_v_1.4

Structural modification able to increase lipophilicity may help to enhance drug potency (Muraglia et al., 2007, 2014). On the other hand, Log *P* > 3 generate concern for possible toxic effects at the cardiac level. As shown in Table 1, the introduction of the tetramethyl-pyrroline moiety raised lipophilicity in acceptable way. This is in line with the observed increase of potency of the new analogs vs. parent compounds. To further understand the molecular bases for this difference, we undertook an *ab initio* molecular modeling study.

**Figure 4:**
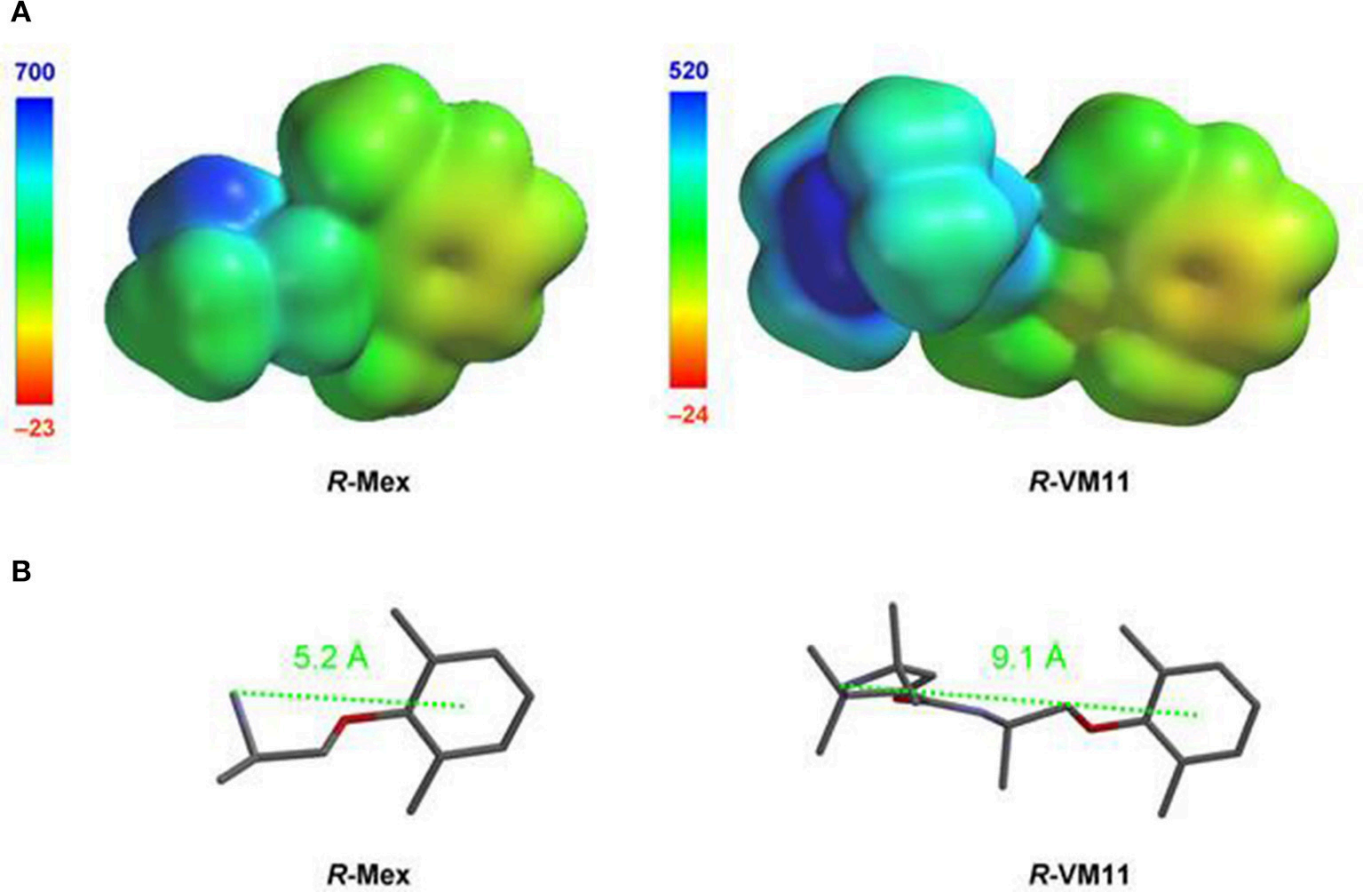
**(A)** Electrostatic potential maps indicating molecular volume and charge distribution of the studied compounds Mex and VM11. The molecular surface regions presenting the most negative electrostatic potential (i.e., the most favorable energy of interaction with a positive charge used as a probe) and the most positive electrostatic potential (i.e., the most unfavorable energy of interaction with the above positive charge used as a probe) were color coded in red and blue false colors, respectively. Thus, warm colors characterize relatively electron-rich regions, with red color indicating the most negative ones. *R*-VM11 electrostatic potential map indicates higher delocalization of the positive charge with its highest potential value (+520 kcalmol^−1^) being lower than the corresponding value in Mex electrostatic potential (+700 kcalmol^−1^). **(B)** Tube representation of the most stable conformer (B3LYP/6-31G**//B3LYP/6-31G**) of R-Mex and R-VM11 (protonated forms).

The tetramethyl-pyrroline basic nitrogen is inserted in a lipophilic structural environment and, when protonated, distributes the cationic charge in the form of partial charges delocalized on the surrounding carbon atoms (Figure 4). We have recently reported that cationic charge delocalization in Mex analogs is beneficial, possibly increasing affinity for Tyr residues (Catalano et al., 2008; De Bellis et al., 2013). Thus, we speculated that replacing primary amine groups of Mex and Me5 with the secondary amine group of tetramethyl-pyrroline ring would reproduce favorable stereolectronic features beneficial for binding. Furthermore, several lines of evidence demonstrate that elongation of the intermediate chain connecting the aryloxy moiety of Mex to the basic functional group improves use-dependency (De Luca et al., 2003a; De Bellis et al., 2013). The introduction of the pyrroline ring by acylation of the nitrogen atom of Mex and its analog reduces to zero the basicity of the formerly protonatable group while introducing a new basic group far from the aryloxy moiety. This structural modification was expected to improve use-dependency of block (Tables 1, 2). The result of this modeling analysis predicts the experimental data well. The latter observation supports the involvement of a strong interaction between the new analogs and the receptor site.

### Cytoprotective profile of tetramethyl-pyrroline derivatives vs. parent compounds

**Figure 5:**
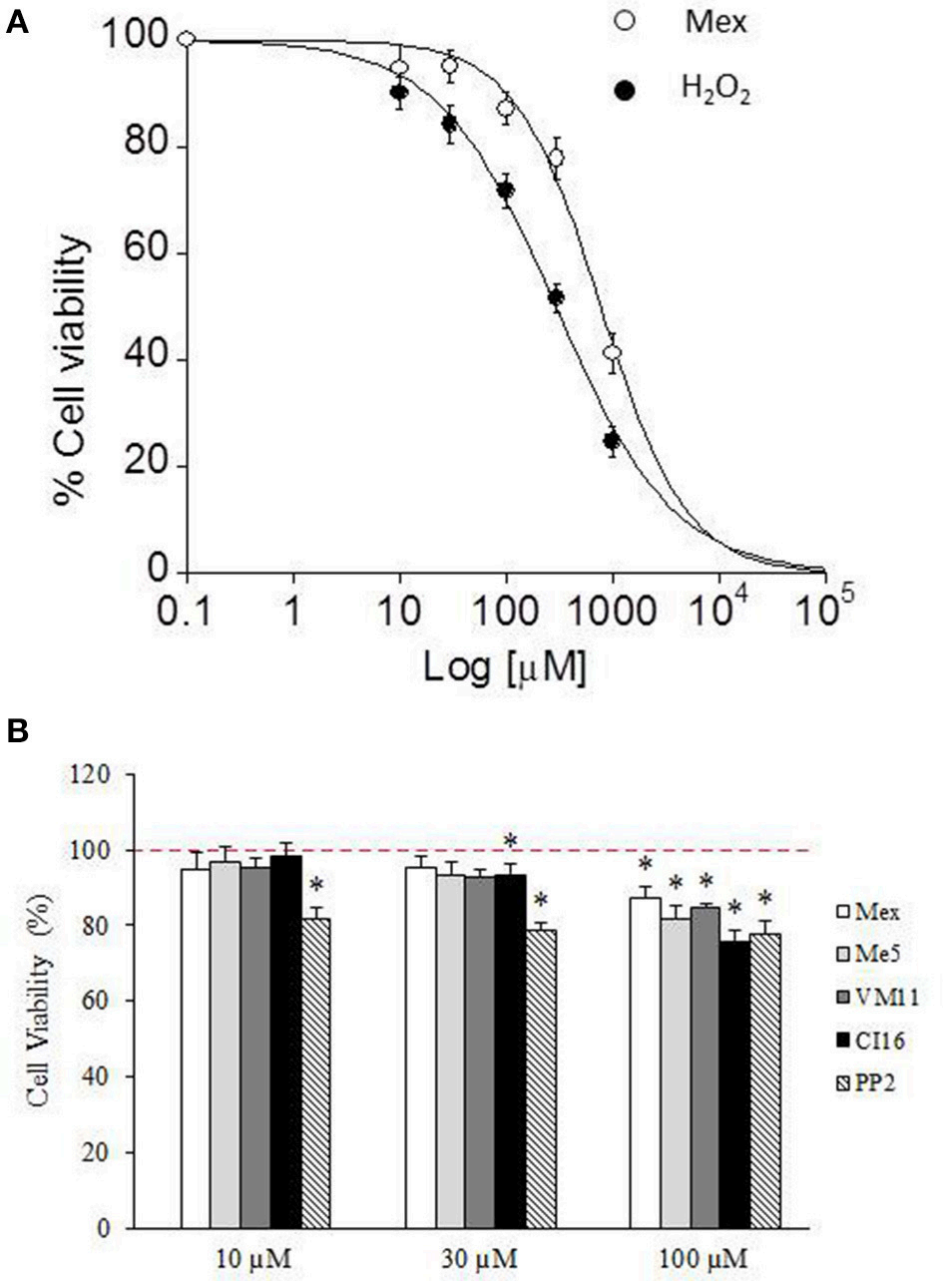
The figure shows the dose-response curve **(A)** of increasing concentration of H_2_O_2_ and mexiletine (10 μM-1 mM) and the cytotoxic effect of increasing concentration (10–100 μM) of mexiletine, its pyrroline derivatives and PP2 **(B)** on cell viability of C_2_C_12_ cells. Values are presented as the mean ± S.E.M. and cell viability (%) is expressed according to the following formula: cell viability (%) = [(test value - blank)/(control value-blank) × 100], where the blank value represents that of cell-free wells, the test value represents that of cells treated with one of the test compounds and the control value represents that of not-treated cells (indicated with dashed line in **B**). Each data is from at least 12–38 replicates (wells) and 3–6 different culture dishes. The statistical significance between groups was evaluated by Student's *t*-test as follow: *significantly different with respect to control value (0.001 < *p* < 0.05).

Another main aim of our study was to investigate about a potential anti-oxidant effect of the tetramethyl-pyrroline-derivatives, by means of assessing their cyto-protective action on a model of H_2_O_2_-induced oxidative stress cytotoxicity. However, data from our and other laboratories underlined a direct potential cytotoxicity of local anesthetic-like drugs on various cell types (Hofmann et al., 2013; Sung et al., 2014; De Bellis et al., 2017a). Then we first evaluated the ability of the lead compound Mex, to modulate by itself C_2_C_12_ cell viability with respect to H_2_O_2_. Both Mex and H_2_O_2_ caused a concentration-dependent decrease in cell viability; although, the curve of Mex was shifted to the right with respect to that of H_2_O_2_, resulting less cytotoxic (Figure 5A). Importantly, Mex exerted the cytotoxic effect with an IC_50_ value (IC_50_ 775.4 ± 88 μM) that was nearly 10 times higher than that necessary to exert a tonic block of Nav1.4 channels, suggesting a modest role of cell cytotoxicity in the therapeutic range. The IC_50_ of H_2_O_2_ was however markedly lower, being 292 ± 25 μM.

**Figure 6:**
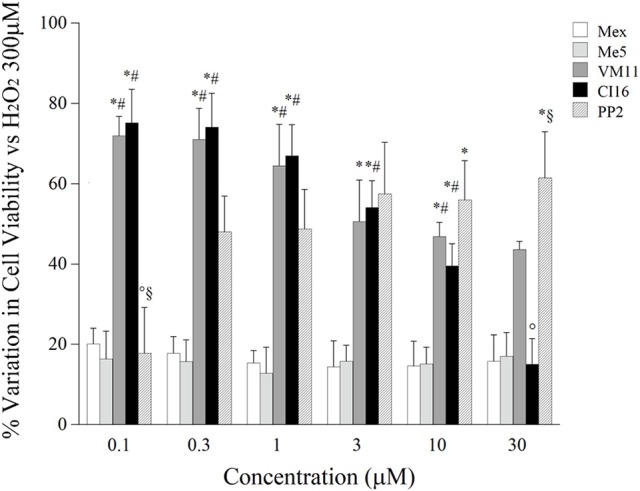
The histograms show the potential cytoprotective effect of increasing concentration of mexiletine, its pyrroline derivatives and PP2 (0.1–30 μM) on cell viability of C_2_C_12_ cells. Values are presented as the mean ± S.E.M. and are expressed as the percentage variation of the control according to the following formula: cell viability (%) = [(test value-blank)/(control value-blank)−1] × 100, where the blank value represents that of a cell-free wells, the test value represents that of cells treated either with one of test compounds or H_2_O_2_ 300 μM and the control value represents that of cells treated with H_2_O_2_ 300 μM alone. Each data is from at least 12–32 replicates (wells) and 3–8 different culture dishes. All test compounds show a significant cyto-protection with respect to the control value. The statistical significance between groups was found by ANOVA analysis (*F* > 5; *p* < 0.03). Bonferroni post-hoc correction for individual differences between groups is as follows: significantly different with respect to *mexiletine (0.0002 < *p* < 0.008); ^#^Me5 (0.0002 < *p* < 0.008); ° VM11 (0.001 < *p* < 0.007) and ^§^CI16 (0.0002 < *p* < 0.0007).

Subsequently, we compared the cytotoxic effect of all the compounds of interest with respect to Mex, in the therapeutic concentration range, i.e., at the concentration where a relevant block of sodium channel occurs (10, 30, and 100 μM). As it can be seen in Figure 5B, up to 30 μM, all the compounds were little if any cytotoxic; while, at 100 μM it is possible to observe a cytotoxicity of the compounds, amounting of about 20%. Interestingly, PP2 was more cytotoxic than Mex and its analogs. Considering that this effect could bias the estimation of cyto-protection, due to a possible synergic action with H_2_O_2_, we attempted to focus the pure antioxidant effect, by assessing the potential cyto-protections against H_2_O_2_ damage only in a concentration range (from 0.1 to 30 μM), at which tonic and use-dependent block of sodium currents did occur (Figure 6). Drugs were incubated 2 h before the application of H_2_O_2_ at 300 μM and 1 mM, as a model for moderate and severe oxidative-stress induced cytotoxicity respectively, and then maintained for additional 6 h for a total incubation of 8 h. Mex and Me5 showed only a moderate, albeit significant, cyto-protective effect in the presence of 300 μM H_2_O_2_. Interestingly, the tetramethyl-pyrroline derivatives showed in the concentration range where a relevant use-dependent block of sodium channel occurs (from 0.1 to 10 μM) a remarkable cyto-protection in C_2_C_12_-myoblasts against H_2_O_2_-dependent-damage, resulting significantly more potent than their parent compounds. The protective effect was still clearly detectable for both compounds; in particular, VM11 maintained the same cyto-protection observed between 3 and 30 μM, while a decrease in efficacy was found with CI16 (Figure 6). VM11 maintained the cyto-protection also at 100 μM, with a 43.5 ± 2.03% of cell protection. The compounds had a similar behavior of other anti-oxidant molecule PP2 (Figure 6) and were more potent that the classical anti-oxidant NAC (Figure S4). For all compounds, no protection was observed at highest H_2_O_2_ concentration (1 mM) exerting more than 80% of cell deaths (data not shown).

## Discussion

The present study was the first attempt to characterize Mex derivatives for their ability to combine the block of voltage-gated sodium channels with an anti-oxidant activity in skeletal muscle, considering the potential need to reduce the stress of unbalanced excitation-contraction in degenerating myopathies in which both alteration of voltage-gated sodium channels and oxidative stress can occur. The large number of physiological processes regulated by sodium channels and their role in many diseases make the voltage-gated sodium channels highly interesting as targets for new drugs (Chahine and Desaphy, 2016; De Bellis et al., 2017b). Mexiletine has been used for two decades as off-label drug in nondystrophic myotonias, and recently has appointed an orphan drug designation in myotonic syndromes. By blocking skeletal muscle sodium channels, mexiletine can counteract sarcolemma over-excitability and alleviate symptoms, whatever myotonia is caused by sodium or chloride channel mutations. In addition, the use of mexiletine is characterized by several drawbacks including epigastric discomfort, atrioventricular heart block, and CNS disturbances. Hence, it has now been discontinued in many countries including the US and the UK (Suetterlin et al., 2015; Roselli et al., 2016). Consequently, numerous attempts have been made in recent years to develop an alternative to mexiletine, including the design of new analogs that offer the same pharmacological effect but without the unwanted side effects (Roselli et al., 2016; De Bellis et al., 2017b). In fact, in a series of our previous structure-activity relationship studies (SAR), we have tested a number of rationally-designed mexiletine derivatives in the attempt to increase potency and use-dependent behavior. Data and molecular modeling show that increasing basicity and optimal alkyl chain length, along with proper substituents on the chiral center, enhance use-dependent block in adult native muscle fibers, leading to more effective antimyotonic agents (De Luca et al., 1997b, 2004; De Bellis et al., 2013). Use-dependent blockers of Na_v_1.4 may be of interest in other myopathic condition, i.e., to control abnormal muscle hyperexcitability and spontaneous discharges of dystrophic subjects without impairing excitation-contraction coupling (De Luca et al., 1997a; Emeryk-Szajewska and Kopeć, 2008; Hirn et al., 2008; Koenig et al., 2011).

Interestingly, various evidences support a potential anti-oxidant and neuroprotective effect of mexiletine in different conditions such as cerebral ischemia, spinal- and head-trauma. In particular, it has been shown that the tetramethyl-pyrroline derivative of Mex and its *in vivo* nitroxide metabolite exhibit protection against reperfusion injury, presumably due to a combination of antioxidant and membrane stabilizing mechanisms. Part of this anti-oxidant activity has been attributed to the pyrroline ring (Shankar et al., 2000).

In this frame, a main finding of the present work was that the introduction of a tetramethyl-pyrroline moiety on the amino group of Mex (as in VM11) and of its potent derivative Me5 (as in CI16), lead to a marked increase in the potency on skeletal muscle voltage-gated sodium channels, especially as use-dependent channel blockers. In particular, CI16 was up to 40-fold more potent to block Na_V_1.4 channels in a use-dependent manner with respect to Mex and it is the strongest use-dependent Mex-like compound described so far. The key structural features of CI16 with respect to the parent compound, i.e., the introduction of a tetramethyl-pyrroline moiety on the amino group of the isopropyl derivative, is a modification that may further enhance the interaction with amino acid residues and/or better positioning of the drug in the channel pore during voltage-dependent conformational changes of Na_V_1.4 (Sheets et al., 2010; De Bellis et al., 2017b), as also supported by the molecular modeling. In fact, the new pyrroline-derivatives maintain the main mode of action of parent compounds in terms of acting with a modest stereo-selective behavior (De Luca et al., 2000; Talon et al., 2001; Muraglia et al., 2007). In addition, the introduction of the pyrroline ring reduces to zero the basicity of the formerly amino group while introducing a new basic group far from the stereogenic center. This structural modification is in line with a further reduction of stereoselectivity since one of the main pharmacophoric elements (the protonatable nitrogen atom) is now far from the stereogenic center. Both CI16 and VM11 appear to have a high affinity for both resting and inactivated channels, the high use-dependent behavior being mostly due to a slow unbinding from the channel and to a favorable change in basicity and logD. This feature makes CI16 a lead compound for controlling abnormal muscle excitability and may deserve *in vivo* testing as antimyotonic drugs.

In addition, and in agreement with the working hypothesis, in the low range of concentrations effective in blocking sodium channels, all the compounds showed a remarkable cyto-protection of C_2_C_12_-myoblasts against H_2_O_2_-dependent-damage, resulting significantly more potent than their parent compounds. Both pyrroline derivatives exerted a cyto-protective action similar and even higher to those observed with compounds of interest for muscular dystrophy, i.e., NAC and PP2. In fact, NAC has been found to reduce markers of oxidative stress in myofibers and to ameliorate primary endpoints of pathology in dystrophic mdx mouse model (Grounds et al., 2008; Allen et al., 2016); accordingly, it has also been proposed for clinical trials in DMD. Similarly, PP2 acts as an inhibitor of cSrc tyrosine kinase, a redox activated enzyme that is overexpressed in dystrophic muscle, and that reinforces oxidative stress damage via activation of NADPH oxidase, the main source of superoxide anion in dystrophic myofibers (Bedard and Krause, 2007; Burdi et al., 2009; Camerino et al., 2014; Capogrosso et al., 2017).

**Figure 7:**
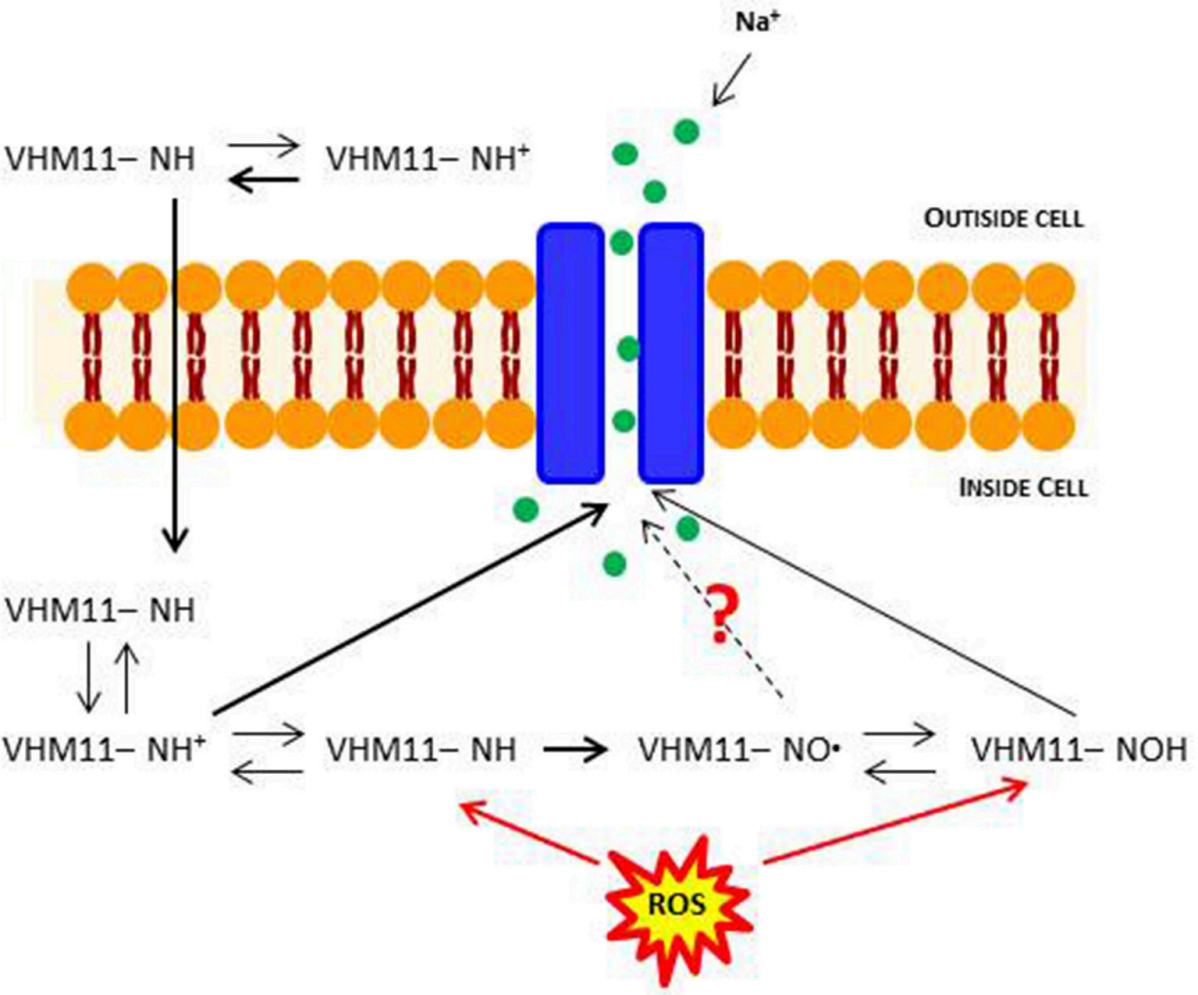
Schematic depictions of the mechanism of block of skeletal muscle sodium channel by local anesthetic drugs (LA*s*), in conditions of oxidative stress. LAs are weak bases and are usually formulated as the hydrochloride salt to render them water-soluble. At a pH equal to the protonated base's p*Ka*, the protonated (ionized) and unprotonated (unionized) forms of the molecule exist in equimolar amounts, but only the unprotonated base diffuses readily across cell membranes. Once inside the cell, the local anesthetic will be in equilibrium, with the formation of the protonated (ionized form), which does not readily pass back out of the cell. This is referred to as “ion-trapping.” In the protonated form, the molecule binds to the LA binding site on the inside of the ion channel. Probably, an excess of ROS could promote molecular mechanisms of sodium channel regulation.

These results pave the ways to more addressed experimental approaches to better assess the mechanism of action underlying the potential anti-oxidant action of the Mex derivatives. In fact, we have chosen H_2_O_2_ to induce an oxidative cell damage in our cultures because it can enter the cells and induce cytotoxicity; also, it can be generated from nearly all sources during oxidative stress and acts as precursor of highly oxidizing, tissue-damaging radicals, such as hydroxyl radicals (Mahakunakorn et al., 2003). However, the effects of cyto-protective compounds on oxidative stress damage is often more complex than expected due to various mechanisms of action and to the multiplicity of pathways involved (Burdi et al., 2006; Pierno et al., 2014; Capogrosso et al., 2016). For instance, *in vitro* and *in vivo* anti-oxidant mechanisms of ionized compounds, such as Mex and its derivatives, may differ, i.e., due to cell penetration issues or to additional effects on pathways different from those responsible for H_2_O_2_ cell damage. A key open question in relation to the present compounds is the exclusivity of action as channel blockers and cyto-protective agents, since part of this latter mechanism can be related *in vivo* to the oxidation of the pharmacophore NH group into the NO moiety, that in turn is in equilibrium with the related N-OH form (Hankovszky et al., 1986; Li et al., 2000). The assessment of the relative contribution to each form to both pharmacological activities is experimentally challenging, considering both the dynamic equilibrium and the fact that the individual testing of simpler NO-like compounds may only partially help as they do not exactly recapitulate the chemical nature of the original molecules. In addition, we have previously demonstrated that N-OH metabolite of Mex still retains a weak activity as sodium channel blocker (De Bellis et al., 2006). The present data allow us to speculate about both activities being finally related to the same molecule. In fact, the block of sodium channel occurs rapidly, and it is likely related to the NH form. In parallel, the cyto-protective action, occurring at a longer time-scale, is also related to the presence of NH, this latter being oxidized to NO in its action as ROS scavenger. A slightly different profile of VM11 and CI16 has been found, the former being more cyto-protective while CI16 showing a greater activity as use-dependent blocker. Then, although the same molecule may retain, in the same concentration ranges, both activities, these latter can be related to slightly different chemical characteristics. A scheme of such an equilibrium is schematically shown for one of the tested pyrrole derivatives in Figure 7. More detailed investigation would help to better define the pharmacological profile of the new compounds *in vitro* and *in vivo*.

In conclusion, our results support the working hypothesis to enlarge the pharmacological activity of Mex-like compounds with the introduction of the pyrroline ring opening toward novel therapeutic application. Meanwhile, the tested Mex derivatives also show an enhancement of therapeutically relevant use-dependent block of skeletal muscle voltage-gated sodium channels. The data fit well with the knowledge obtained by previous structure activity relationship studies and support the possibility to carry on *in silico* drug design for skeletal muscle sodium channels as successfully done for other ion channels (Wacker et al., 2012; Gobbi et al., 2016). Structure activity investigation are also pivotal to identify compounds with better toxicological profile avoiding off target effects (Tricarico et al., 2002, 2012; Plass et al., 2005).

Since the use-dependence properties selectively address the activity of a sodium channel blocker against pathological conditions, the compounds CI16 and VM11 deserve further investigation of their potency in pathologies characterized by primary or secondary defects of membrane hyperexcitability both *in vitro* and *in vivo*. In particular, only *in vivo* experiments, currently under planning in our laboratories in proper animal models, will allow to finally shed light on the dual mechanism of action of these Mex-derivatives and to better support the interest in combining structural modifications to enlarge the pharmacological profile of sodium channel blockers.

## Ethics statement

The animal care was performed in accordance with the DIRECTIVE 2010/63/EU. The study is, approved by national ethic committee for research animal welfare of the Italian Minister of Health.

## Author contributions

ADL, MDB, and GL elaborated the hypothesis; DCC and ADL designed the studies; GL and AC synthesized the compounds and performed the modeling; MDB and FS conducted the experiments; MDB, SP, and J-FR analyzed the results; MDB, ADL, and J-FR wrote the manuscript and all authors approved the manuscript.

### Conflict of interest statement

The authors declare that the research was conducted in the absence of any commercial or financial relationships that could be construed as a potential conflict of interest.
