# Supplementary material for: Dual Action of Mexiletine and Its Pyrroline Derivatives as Skeletal Muscle Sodium Channel Blockers and Anti-oxidant Compounds: Toward Novel Therapeutic Potential
Source: Front Pharmacol. 2018 Jan 12;8:907. doi: 10.3389/fphar.2017.00907 (PMC5770958; doi:10.3389/fphar.2017.00907)
Supplement: Supplementary file 1 [file DataSheet1.pdf]

SUPPLEMENTARY MATERIALS for:

DUAL ACTION OF MEXILETINE AND ITS PYRROLINE DERIVATIVES AS SKELETAL MUSCLE SODIUM CHANNEL BLOCKERS AND ANTI-OXIDANT COMPOUNDS: TOWARD NOVEL THERAPEUTIC POTENTIAL

De Bellis M., Sanarica F., Carocci A., Lentini G., Pierno S., Rolland J.F., Conte Camerino D., De Luca A.

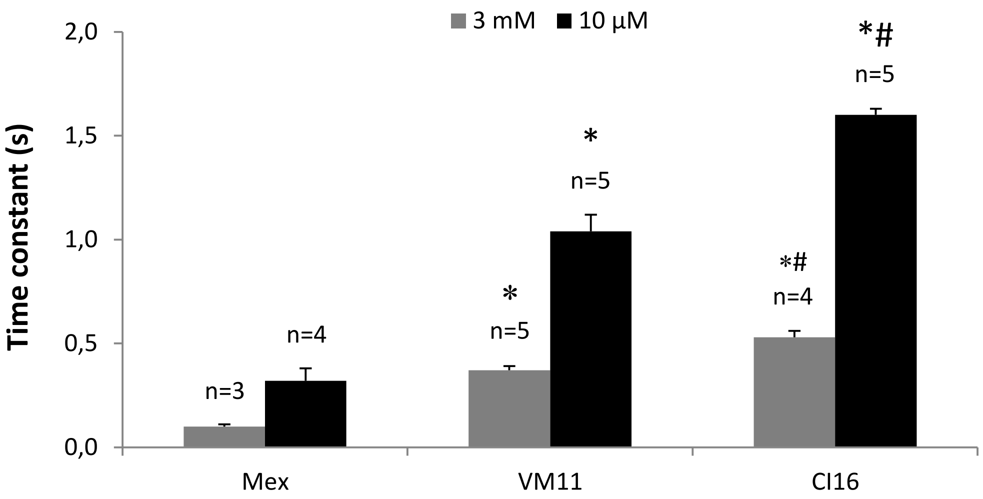

Fig S1: Time constant values for recovery from inactivation of Mex and its tetramethyl-pyrroline derivatives *vs* the concentrations of each compound.

ANOVA tests was significant for both 3 μM and 10 μM with $F > 57$ and $p < 8 \cdot 10^{-6}$. Bonferroni post-hoc correction is as follow: ∗ significantly different with respect to Mex ($3\ 10^{-8} < p < 7.10^{-5}$); # significantly different with respect to VM11 ($5 \cdot 10^{-5} < p < 0.001$).

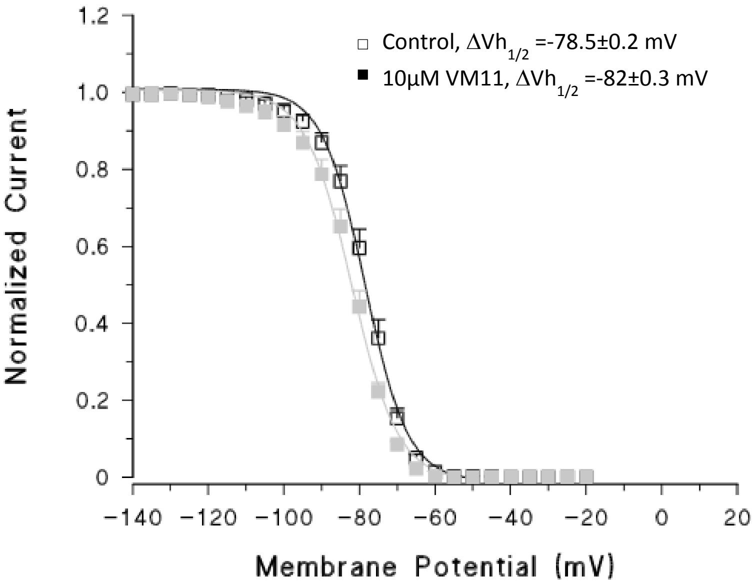

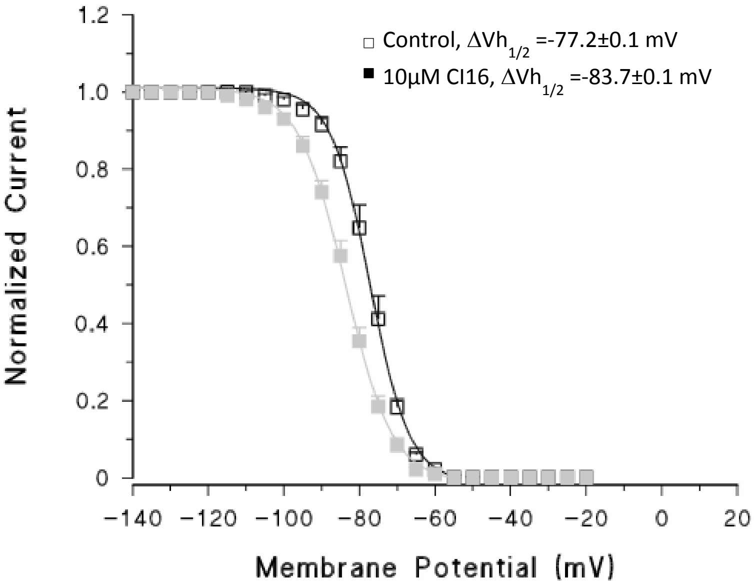

Fig. S2: Steady-state inactivation (h∞) curves were determined by a cyclic protocol of pulse sequences. Each sequence consisted of a conditioning pulse to -140 mV, a prepulse of 1000 ms duration, and a test pulse to -20 mV for 10ms; after a pause of 3 s, the sequence was repeated 18-20 times with the prepulse potential value increased each time in 5 mV steps. A more quantitative estimate of the absolute affinity constant for the inactivated state (K_i) was obtained from the voltage dependent distribution of the channels in the resting (h) and inactivated state (1-h) obtained from the h∞ curve, using the equation $1/K_{-70} = h/K_r\ (1\text{-}h)/K_i$, where K_{-70} and K_r are the IC_{50} values

obtained from dose-response curves at -140 and -70 mV, and h and (1-h) represent the fraction of channels in the resting and inactivated state at -70 mV, respectively.

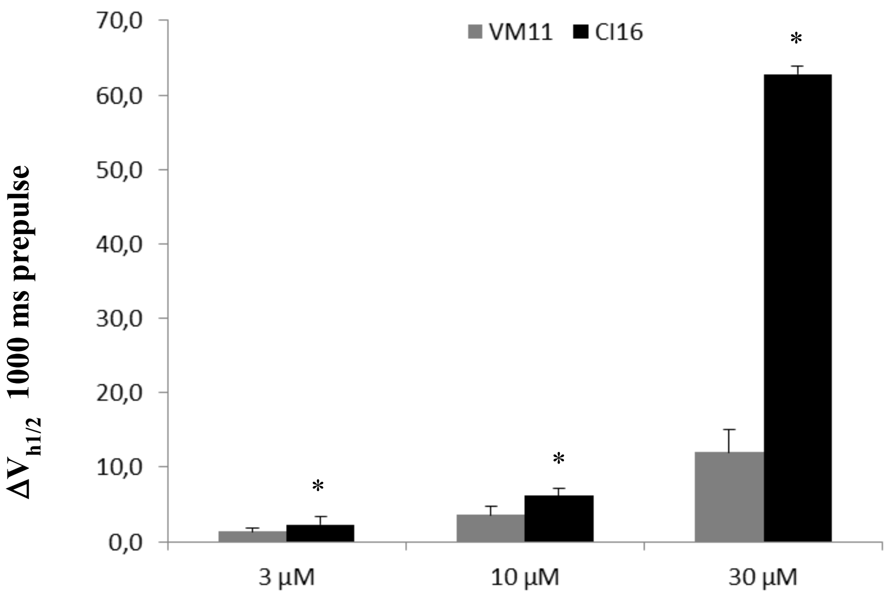

Fig. S3 : Shift of the steady-state inactivation curves, calculated at the $Vh_{1/2}$ value, produced by the test compounds at concentrations of 3, 10 and 30 μM. For each drug, the left shift produced has been calculated in any individual experiments *versus* the related $Vh_{1/2}$ control value in the absence of drug. The individual values have been averaged. Each bar shows the shift of the h_∞ curve produced by each compound and each point is the mean ± S.E.M. of three to six individual determinations.

∗ shows the statistic significance by Student's *t* distribution (for ($p < 0.001$ or less) between the tetramethyl-pyrroline derivatives for each concentration.

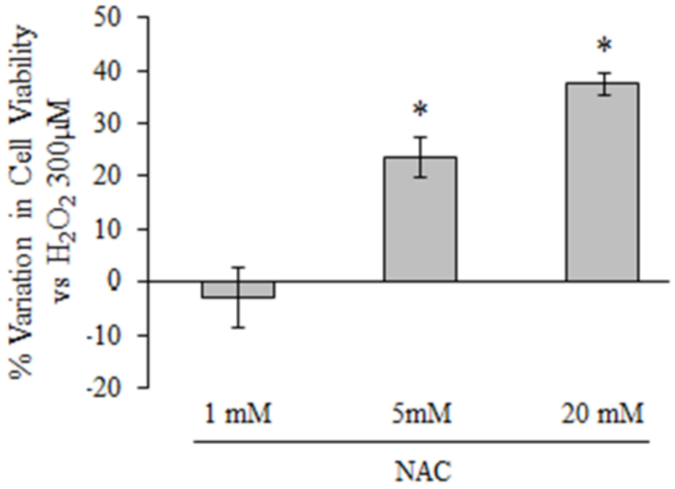

Fig. S4 : Cytoprotective effect of NAC on H_2O_2-induced cytotoxicity. The figure shows the potential cytoprotective effect of increasing concentration of NAC on cell viability. Values are presented as the mean ± SEM and are expressed as the percentage variation in cell viability with respect to 300 µM H_2O_2 according to the following formula: cell viability (%) = [(test value - blank) / (control value - blank) -1] × 100, where the blank value represents that of a cell-free wells; the test value represents that of wells of cells treated with either one of the test compounds or 300 µM H_2O_2 and the control value represents that of wells of cells treated with 300 µM H_2O_2 alone. Each data is from at least 4-24 replicates (wells) and 1-4 different culture dishes. The statistical significance between groups was evaluated by Student's t-test as follow: * significantly different with respect to control value ($0.001<p<0.005$).
